# Large vessel occlusion mediated fluid attenuated inversion recovery signal intensity ratio is associated with stroke within 4.5 h

**DOI:** 10.3389/fneur.2024.1445017

**Published:** 2024-10-07

**Authors:** Yajing Wang, Qianqian Mao, Liang Jiang, Mingyang Peng, Yu-Chen Chen, Hong Zhang, Liwei Wang, Xindao Yin

**Affiliations:** ^1^Department of Radiology, Nanjing First Hospital, Nanjing Medical University, Nanjing, China; ^2^Department of Radiology, Affiliated Jiangning Hospital of Nanjing Medical University, Nanjing, China

**Keywords:** stroke, diffusion-weighted imaging, fluid attenuated inversion recovery, onset time, signal-intensity ratio

## Abstract

**Introduction:**

The primary objective was to investigate the value of the fluid attenuated inversion recovery (FLAIR) signal intensity ratio (SIR) in identifying stroke within 4.5 h. The secondary objective was to ascertain whether large vessel occlusion (LVO) mediated the relationship between the SIR and stroke within 4.5 h.

**Methods:**

We analyzed 633 acute stroke patients within 24 h of clear symptom onset. The SIR and DWI-FLAIR mismatch were evaluated. First, we determined whether demographic variables, vascular risk factors and LVO were related to stroke within 4.5 h with multivariate logistic regression analyses and stratified regression analysis. Next, we used mediation analysis to determine whether LVO explained the association between SIR and stroke within 4.5 h. Finally, we used receiver operating characteristic (ROC) analysis to assess the value of SIR, independent variable, and multiparameter models in identifying stroke within 4.5 h and compared with DWI-FLAIR mismatch.

**Results:**

Hyperlipemia, LVO and SIR were associated with stroke within 4.5 h. Mediation analysis revealed that LVO partially mediated the relationship between SIR and stroke within 4.5 h (*p* < 0.001). The multiparameter model (hyperlipemia, LVO and SIR) showed significantly improved performance (AUC 0.869) in identifying stroke within 4.5 h over DWI-FLAIR mismatch (0.684), hyperlipemia (0.632), LVO (0.667) and SIR (0.773) models.

**Conclusion:**

SIR is associated with stroke within 4.5 h, and LVO partially mediates this relationship. A multiparameter model combining hyperlipemia, LVO and SIR can more accurately identify stroke within 4.5 h than individual parameter models.

## Introduction

Acute ischemic stroke is a highly prevalent cardiovascular disease and ranks as the second leading cause of death worldwide (1, 2). Approximately 15–30% of acute ischemic stroke patients experience wake-up stroke (WUS), characterized by the occurrence of stroke symptoms during sleep (3). The accurate determination of the stroke onset time for WUS patients is highly challenging, and thus their eligibility for early thrombolysis and endovascular treatment is impeded. Consequently, this limitation results in missed opportunities for reperfusion, contributing to the observed high disability rate among WUS patients (4). Additionally, the low survival rates after treating the ischemic penumbra and areas of reperfusion injury further complicates the adverse outcomes associated with this condition (5, 6).

Presently, multiparametric magnetic resonance imaging (MRI) is widely acknowledged as a valuable and noninvasive modality for assessing the onset time (7). Studies have demonstrated that diffusion-weighted imaging (DWI) and fluid attenuated inversion recovery (FLAIR) mismatch is a promising imaging biomarker capable of identifying individuals with acute stroke who fall within the reperfusion treatment timeframe (8, 9). The predictive accuracy of DWI-FLAIR mismatch in determining the onset time of WUS has been demonstrated in previous studies conducted by Taner Bulut et al. (10) (accuracy of 0.72) and Wouters et al. (11) (accuracy of 0.68). However, this method suffers from low sensitivity and low interrater consistency (12). We attempted to identify the onset time using machine learning methods based on DWI and FLAIR (13). However, this methodology requires physicians to manually outline the region of interest (ROI), leading to considerable time expenditures. Consequently, there is an urgent need for an objective and quantitative method for precisely identifying the onset time of WUS using imaging modalities. Quantitative MRI sequence parameters, specifically the signal intensity ratio (SIR), can serve as a viable alternative. The SIR is determined by comparing the signal intensity (SI) of DWI lesions on the corresponding FLAIR images with the average SI of the contralateral normal brain hemisphere. Previous studies have demonstrated that the SIR exhibited greater sensitivity and specificity than did the DWI-FLAIR method in identifying the stroke onset time. Studies conducted by Scheldeman et al. (14) and Legge et al. (15) utilized SIR thresholds to identify onset times surpassing 4.5 h and 6 h, with corresponding sensitivities of 0.77 and 0.86, respectively. However, in addition to the SIR, we assume the time course of acute stroke is associated with vascular risk factors (hypertension, hyperlipidemia, etc.) and large vessel occlusion (LVO). This latter condition may shorten the onset time via the remodeling of the cerebral vasculature, including lowering the vessel caliber and reducing cerebral blood flow (16). However, the impact of these factors, especially LVO, on the SIR has not been fully explored. Furthermore, no study has definitively shown a relationship between the SIR and the onset time, raising the question of whether these risk factors could play a prominent role in this relationship.

In a comprehensive analysis of a large sample of acute stroke patients with known onset times, we aimed to investigate the associations among vascular risk factors, LVO, SIR, and stroke within 4.5 h. In addition, we assessed the value of the SIR and multiple parameters in identifying patients within 4.5 h of stroke onset and compared the results with those of patients with DWI-FLAIR mismatch. Previous studies (17, 18) have shown that vascular risk factors (such as hypertension, hyperlipidemia, etc.) are independent risk factors for acute stroke. These patients are more likely to cause vascular inflammatory changes or lipid deposition (19), leading to vascular occlusion and insufficient blood supply to brain tissue, accelerating hypoxia necrosis of surrounding brain cells, and thus more likely to experience changes and clinical symptoms of cerebral infarction. So we hypothesized that the vascular risk factors would be related to the stroke onset time. We also hypothesized that LVO would be related to SIR and that LVO plays a key role in mediating the relationship between SIR and stroke within 4.5 h.

## Methods

### Study population

The study retrospectively incorporated data from Nanjing First Hospital and the Affiliated Jiangning Hospital of Nanjing Medical University from January 2017 to September 2023. Patients were included if they had anterior circulation acute ischemic stroke and a clear stroke symptom onset time within 24 h and if they had undergone MRI scans before therapy. All acute stroke patients generally undergo MRI examination upon admission, unless the patient has contraindications to MRI examination (such as claustrophobia or cerebral hemorrhage). A total of 678 patients were considered candidates for analysis; 45 individuals had significantly distorted DWI or FLAIR images. Ultimately, a total of 633 patients were included in the analysis. The hospital review board of Nanjing Medical University approved the study protocol. All patients in this study provided written informed consent before the MRI examination. Given the retrospective design of this study, the requirement for obtaining informed consent from participants was waived. The onset time is defined as the interval between the initial presentation of stroke symptoms and the subsequent MRI examination in patients. The patients were divided into two groups according to the onset time: positive (≤4.5 h) and negative (>4.5 h).

### Risk factors

Age, sex, National Institutes of Health Stroke Scale (NIHSS) score on admission, and any history of hypertension, diabetes, hyperlipidemia, smoking, drinking or atrial fibrillation were recorded.

### MRI protocol

MRI data were obtained using a Siemens 3.0 T MRI scanner (Magnetom Prisma, Siemens Healthineer). The MRI protocol included DWI, FLAIR, magnetic resonance angiography (MRA) and perfusion-weighted imaging (PWI) sequences. DWI: spin-echo (SE) sequence; repetition time (TR), 2,600 ms; echo time (TE), 98 ms; acquisition matrix, 224 × 224; field of view (FOV), 220 × 220 mm; flip angle (FA), 90°; slices, 16; slice thickness, 6 mm; intersection gap, 1.3 mm; b values, 0 and 1,000 s/mm^2^. FLAIR: inversion recovery (IR) sequence; TR, 7,000 ms; TE, 120 ms; acquisition matrix, 356 × 151; FOV, 230 mm × 230 mm; FA, 90°; slices, 18; section thickness, 5 mm; intersection gap, 0 mm. MRA: gradient recalled echo (GRE) sequence; TR, 20 ms; TE, 3.3 ms; acquisition matrix, 528 × 531; FOV, 220 × 220 mm; slice thickness, 0.60 mm. PWI: T2*-weighted gradient recalled echo (T2*GRE) sequence; TR, 1,500 ms; TE, 30 ms; acquisition matrix, 128 × 128; FOV, 220 × 220 mm; FA, 90°; slice thickness, 4 mm. Fifty phases and 20 images per phase were obtained. During dynamic acquisition, a 0.1 mmol/kg dose of contrast agent (Magnevist, Bayer Schering Pharma) was injected at a rate of 4 mL/s.

### Imaging analysis

#### Quantitative analysis

**Figure 1:**
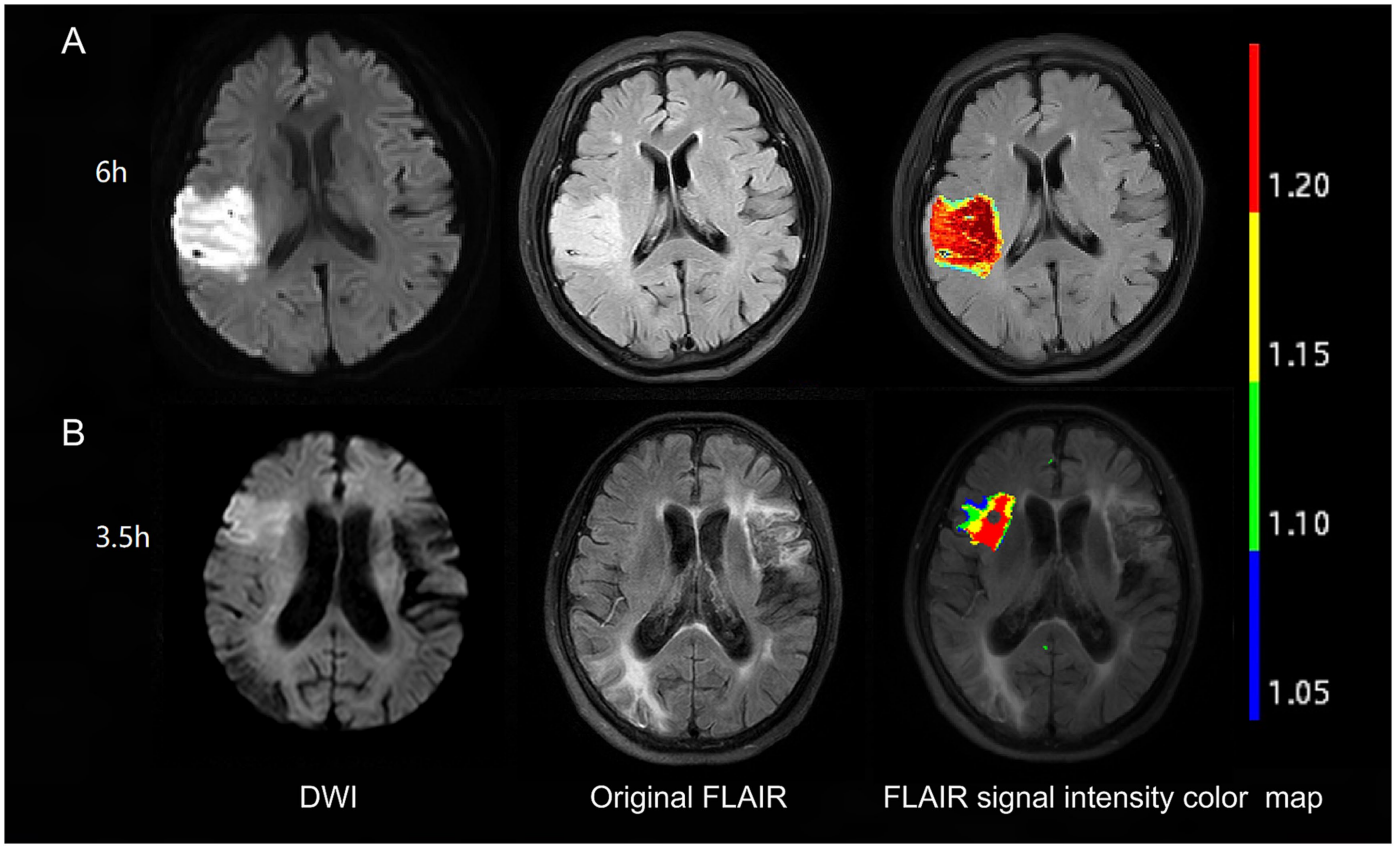
Examples of FLAIR signal intensity color maps in two patients. Patient with 6 h from symptom onset to MRI time (A) has no DWI-FLAIR mismatch with FLAIR SIR of 1.25. Patient with 3.5 h from symptom onset to MRI time (B) has DWI-FLAIR mismatch with FLAIR SIR of 1.13. FLAIR, fluid-attenuated inversion recovery; DWI, diffusion-weighted imaging; SIR, signal-intensity ratio.

NeuBrainCARE software was used to automatically delineate ROIs on DWI [apparent diffusion coefficient (ADC) value <600 × 10^−6^ mm^2^/s]. Subsequently, we matched the DWI and FLAIR images, transferring the ROI delineated on DWI to the FLAIR. The FLAIR SI in this transferred ROI was extracted and compared to the corresponding FLAIR SI in the contralateral normal brain hemisphere. The FLAIR SIR was calculated by dividing the signal value in the corresponding region in the FLAIR sequence of the ischemic lesion on the DWI slice by the FLAIR signal value in the normal opposite side of the cerebrum at the same level (Figure 1).

#### Qualitative analysis

The equivalent of the lesion observed in the DWI sequence was evaluated visually in the FLAIR sequence. The presence of an area with a DWI-positive and FLAIR-negative signal was defined as DWI-FLAIR mismatch (Figure 1). Two independent raters (MP and HZ) visually judged the presence of the DWI/FLAIR mismatch. Images whose assessments were discordant between these two observers were analyzed by a third reader with more than 20 years of MRI reading experience (XY), and this final evaluation was used to evaluate the visual analysis results.

### Statistical analysis

The R software package (version 4.0.3) was used to perform the statistical analyses. The Kolmogorov–Smirnov test was used to assess the normality of continuous variables. Continuous variables are presented as medians (interquartile ranges) and were assessed by student’s *t*-tests and Mann–Whitney *U* tests. Categorical variables are presented as percentages and were assessed by the *χ*^2^ test. Univariate and multivariate logistic regression analyses were used to evaluate the association between the SIR and clinical risk factors and stroke within 4.5 h. A analysis was done with logistic regression with the stratification factors as covariates to evaluate their predictive value. Furthermore, Spearman’s correlation test was used to evaluate the correlations between the SIR value and the onset time with or without LVO. Mediation analysis was conducted to test whether LVO was a mediator of SIR with the onset time. The statistical significance of the mediation effect was analyzed using the R package “mediation” (version 4.5.0). Finally, receiver operating characteristic (ROC) curve analysis was performed to assess the performance of models based on significant variables, the SIR, DWI-FLAIR mismatch and multiple parameters in identifying stroke within 4.5 h. The area under the curve (AUC), sensitivity, specificity, negative predictive value and positive predictive value were used to evaluate the performance of the models. The differences in performance revealed by ROC curve analysis were evaluated using Delong test. All statistical tests were two-sided, and *p*-values <0.05 were considered to indicate statistical significance.

## Results

### Characteristics of the study population

Of the 633 acute stroke patients, 358 underwent MRI within 4.5 h of symptom onset, and 275 underwent MRI more than 4.5 h. In this study, the age of the patients ranged from 27 to 94 years, and the mean age was 68.66 (61, 79) years. The detailed characteristics are presented in Table 1. Compared with patients in the symptom onset to MRI time ≤4.5 h group, patients with time >4.5 h group had a greater prevalence of hyperlipemia (35.6% vs. 9.2%; *p* < 0.001), almost double the prevalence of LVO (64.4% vs. 31.0%; p < 0.001), higher SIR values [1.17 (1.13, 1.22) vs. 1.1 (1.08, 1.15); *p* < 0.001], and lower DWI-FLAIR mismatch rates (20.4% vs. 57.3%; *p* < 0.001).

**Table 1:** Baseline characteristics comparison between symptom onset to MRI time £ 4.5 h and symptom onset to MRI time >4.5 h.

Characteristics	Symptom onset to MRI time £ 4.5 h (*n* = 358)	Symptom onset to MRI time >4.5 h (*n* = 275)	*p*-value
Sex, *n* (%)	233 (65.1%)	192 (69.8%)	0.209
Age, median (IQR)	71 (62, 79)	69 (59, 78)	0.137
NIHSS score on admission, median (IQR)	8 (3, 13)	9 (4, 13)	0.288
Hypertension, *n* (%)	275 (76.8%)	220 (80.0%)	0.336
Diabetes, *n* (%)	111 (31.0%)	99 (36.0%)	0.186
Hyperlipemia, *n* (%)	33 (9.2%)	98 (35.6%)	**<0.001**
Smoking, *n* (%)	100 (27.9%)	64 (23.3%)	0.185
Drinking, *n* (%)	68 (19.0%)	46 (16.7%)	0.462
Atrial fibrillation, *n* (%)	105 (29.3%)	68 (24.7%)	0.198
Symptom onset to MRI time, h (IQR)	3.5 (1.5, 4)	7.5 (6, 12)	**<0.001**
PWI/DWI mismatch, median (IQR)	2.0742 (1.2333, 5.6396)	2.5051 (1.8, 6.2495)	0.067
Large artery occlusion, *n* (%)	111 (31.0%)	177 (64.4%)	**<0.001**
DWI volume, mL	14 (5, 28)	17 (5, 31)	0.053
SIR, median (IQR)	1.1 (1.08, 1.15)	1.17 (1.13, 1.22)	**<0.001**
DWI-FLAIR mismatch, *n* (%)	205 (57.3%)	56 (20.4%)	**<0.001**

Numbers are *n* (%) or median (interquartile ranges), as appropriate. NIHSS, National Institutes of Health Stroke Scale; PWI, perfusion-weighted imaging; DWI, diffusion-weighted imaging; SIR, signal intensity ratio. FLAIR, fluid attenuated inversion recovery; LVO, large vessel occlusion. Bold values are used to emphasize the statistical significance of variables.

### Logistic regression analysis

According to univariate regression analysis, hyperlipemia [OR (95% CI): 5.453 (3.530–8.423); *p* < 0.001], LVO [OR (95% CI): 4.019 (2.880–5.609); *p* < 0.001] and the SIR [OR (95% CI): 609562.476 (35789.894–10381880.832); *p* < 0.001] were associated with stroke >4.5 h. After stepwise multiple logistic regression analysis, hyperlipemia [OR (95% CI): 5.615 (3.471–9.082); *p* < 0.001], LVO [OR (95% CI): 2.889 (1.973–4.231); *p* < 0.001] and the SIR [OR (95% CI): 73689.881 (3873.296–1401958.065); *p* < 0.001] remained independently associated with stroke >4.5 h. Table 2 summarizes the results of the logistic regression analyses.

**Table 2:** Univariate and multivariate regression analysis of risks in identifying the time from stroke onset.

Characteristics	Univariate analysis		Multivariate analysis	
	Odds ratio (95% CI)	*p*-value	Odds ratio (95% CI)	*p*-value
Sex	1.241 (0.886–1.738)	0.209		
Age	0.991 (0.979–1.003)	0.158		
NIHSS score on admission	1.011 (0.989–1.034)	0.322		
Hypertension	0.828 (0.564–1.216)	0.337		
Diabetes	0.799 (0.573–1.114)	0.186		
Hyperlipemia	5.453 (3.530–8.423)	**<0.001**	5.615 (3.471–9.082)	**<0.001**
Smoking	0.783 (0.545–1.125)	0.185		
Drinking	0.857 (0.567–1.294)	0.462		
Atrial fibrillation	1.263 (0.885–1.804)	0.198		
PWI/DWI mismatch	1.000 (0.999–1.002)	0.979		
Large artery occlusion	4.019 (2.880–5.609)	**<0.001**	2.889 (1.973–4.231)	**<0.001**
SIR	3.450 (1.497–5.341)	**<0.001**	6.438 (3.281–8.453)	**<0.001**

NIHSS, National Institutes of Health Stroke Scale; PWI, perfusion-weighted imaging; DWI, diffusion-weighted imaging; SIR, signal intensity ratio. Bold values are used to emphasize the statistical significance of variables.

### Stratification regression model

Stratification regression model were performed to further evaluate the ability of hyperlipemia, LVO and the SIR to identify stroke within 4.5 h. The first stratification, which included demographic variables and stroke risk factors as covariates, showed that hyperlipemia was associated with stroke within 4.5 h. We then added PWI/DWI mismatch, the most commonly used imaging parameter, to this regression model. As predicted, we did not observe significant changes in model performance (Δ*R*^2^ = 0; Δ*F* value: *F* = 0.083, *p* = 0.773). After stepwise addition of LVO and the SIR to the model, the results showed that the inclusion of LVO increased the adjusted *R*^2^ of the model (Δ*R*^2^ = 0.094), and a likelihood ratio test confirmed that the full model was an improvement over the original model (Δ*F* value: *F* = 74.741, *p* = 0.000). The SIR value further increased the adjusted *R*^2^ of the model (Δ*R*^2^ = 0.083), and the full model showed further improvements in identification performance (Δ*F* value: *F* = 73.076, *p* = 0.000) (Table 3).

**Table 3:** Stratification analysis of risks in identifying the time from stroke onset.

	Stratification 1	Stratification 2	Stratification 3	Stratification 4
	B ± SE	B ± SE	B ± SE	B ± SE
Sex	0.070 ± 0.044	0.07 ± 0.44	0.05 ± 0.041	0.028 ± 0.039
Age	−0.002 ± 0.002	−0.002 ± 0.002	−0.002 ± 0.002	−0.003 ± 0.002*
NIHSS score on admission	0.004 ± 0.003	0.004 ± 0.003	−0.004 ± 0.003	−0.003 ± 0.003
Hypertension	0.06 ± 0.047	0.059 ± 0.047	0.052 ± 0.045	0.053 ± 0.042
Diabetes	0.006 ± 0.041	0.005 ± 0.041	0.016 ± 0.039	0.011 ± 0.037
Hyperlipemia	0.396 ± 0.047^**^	0.396 ± 0.047^**^	0.361 ± 0.044^**^	0.325 ± 0.042^**^
Smoking	−0.108 ± 0.062	−0.107 ± 0.062	−0.078 ± 0.059	−0.084 ± 0.055
Drinking	−0.02 ± 0.068	−0.021 ± 0.068	−0.046 ± 0.065	−0.049 ± 0.061
Atrial fibrillation	−0.054 ± 0.046	−0.054 ± 0.046	−0.051 ± 0.043	−0.044 ± 0.041
PWI/DWI mismatch		0	0	0
Large artery occlusion			0.329 ± 0.038^**^	0.232 ± 0.038^**^
SIR				1.790 ± 0.209^**^
*R* ^2^	0.123	0.123	0.217	0.3
Adjusted *R*^2^	0.11	0.109	0.203	0.286
*F* value	*F* (9, 623) = 9.704, *p* = 0.000	*F* (10, 622) = 8.729, *p* = 0.000	*F* (11, 621) = 15.671, *p* = 0.000	*F* (12, 620) = 22.122, *p* = 0.000
Δ*R*^2^	0.123	0	0.094	0.083
Δ*F* value	F (9, 623) = 9.704, *p* = 0.000	*F* (1, 622) = 0.083, P = 0.773	*F* (1, 621) = 74.741, *p* = 0.000	*F* (1, 620) = 73.076, p = 0.000
	^*^p < 0.05 and ^**^p < 0.01			

NIHSS, National Institutes of Health Stroke Scale; PWI, perfusion-weighted imaging; DWI, diffusion-weighted imaging; SIR, signal intensity ratio.

### Correlation analysis and mediation analysis

**Figure 2:**
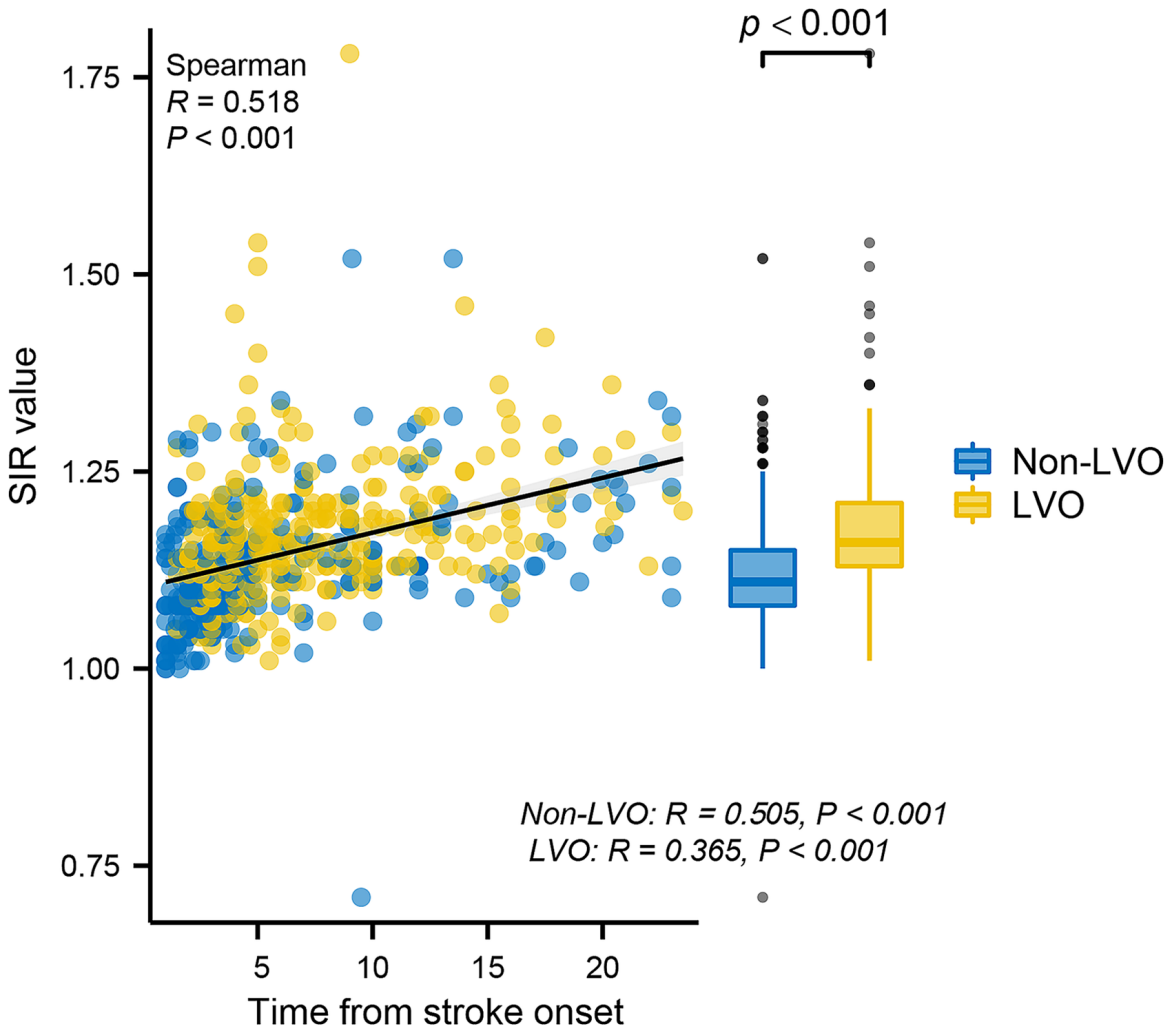
Scatterplot representing the correlations between the SIR and time from stroke onset. The SIR was positively correlated with the time from stroke onset in all patients, patients without LVO and patients with LVO. In addition, there was a significant difference in the SIR between the non-LVO group and the LVO group. SIR, signal-intensity ratio; LVO, large vessel occlusion.

**Figure 3:**
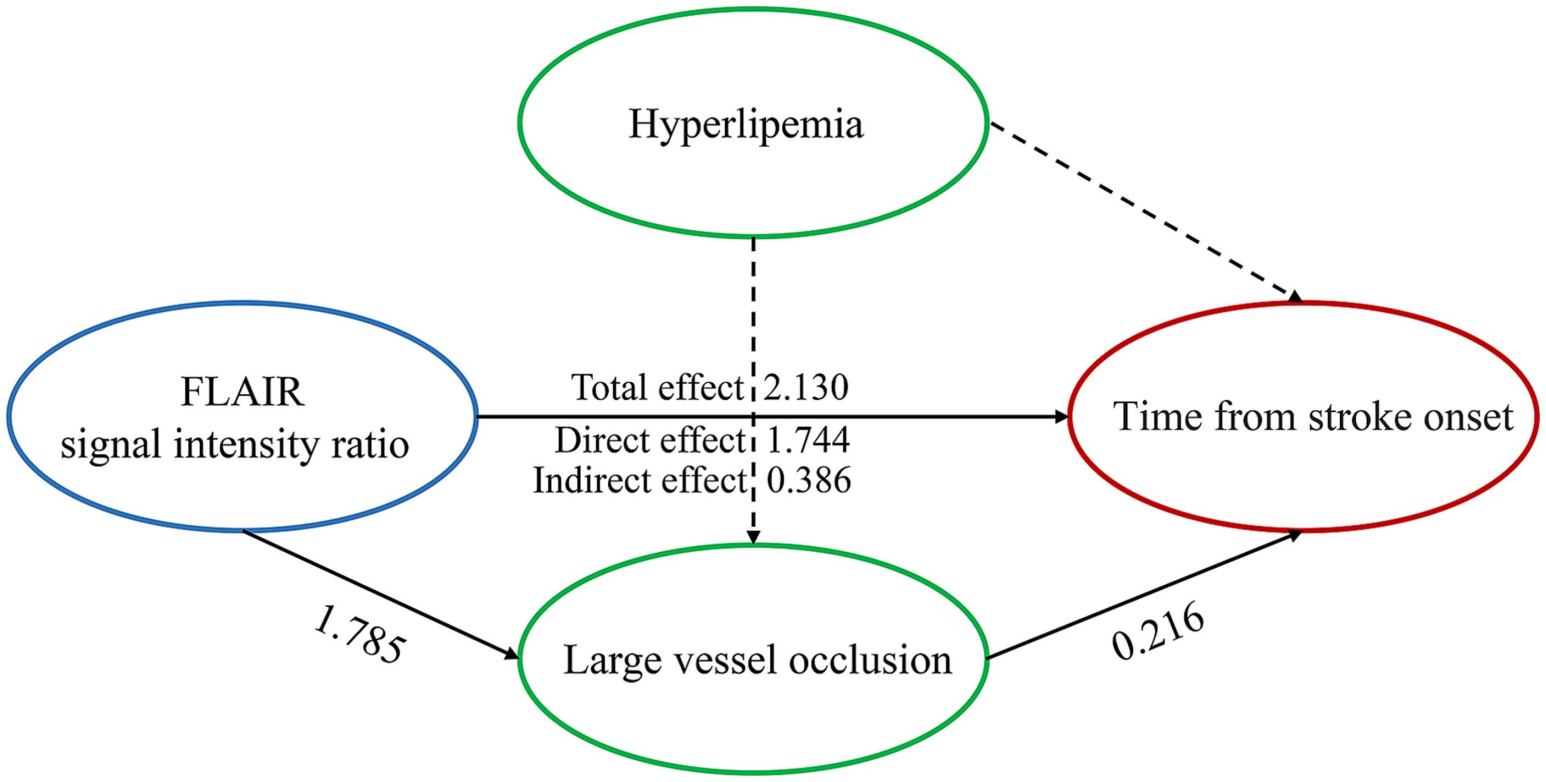
Large vessel occlusion partially mediates the relationship between the FLAIR signal intensity ratio and the time from stroke onset when hyperlipemia is used as a control variable. All paths were significant at *p* < 0.001. Control variables are presented with dashed lines.

Spearman correlation analysis revealed that the SIR was positively correlated with the time from symptom onset to MRI in all patients (*R* = 0.518; *p* < 0.001), patients without LVO (*R* = 0.505; *p* < 0.001) and patients with LVO (*R* = 0.365; *p* < 0.001) (Figure 2), and the Figure 2 also indicates a significant difference in the SIR between the patients without LVO and LVO (*p* < 0.001). The results of the mediation analysis are shown in Figure 3. All regression pathways were significant (*p* < 0.001). We included hyperlipemia as a control variable in the model when estimating the mediating effect of LVO on SIR and onset time. Mediation analysis revealed that the relationship between SIR and stroke within 4.5 h was partially mediated by LVO (*p* < 0.001).

### ROC analysis of variables

**Figure 4:**
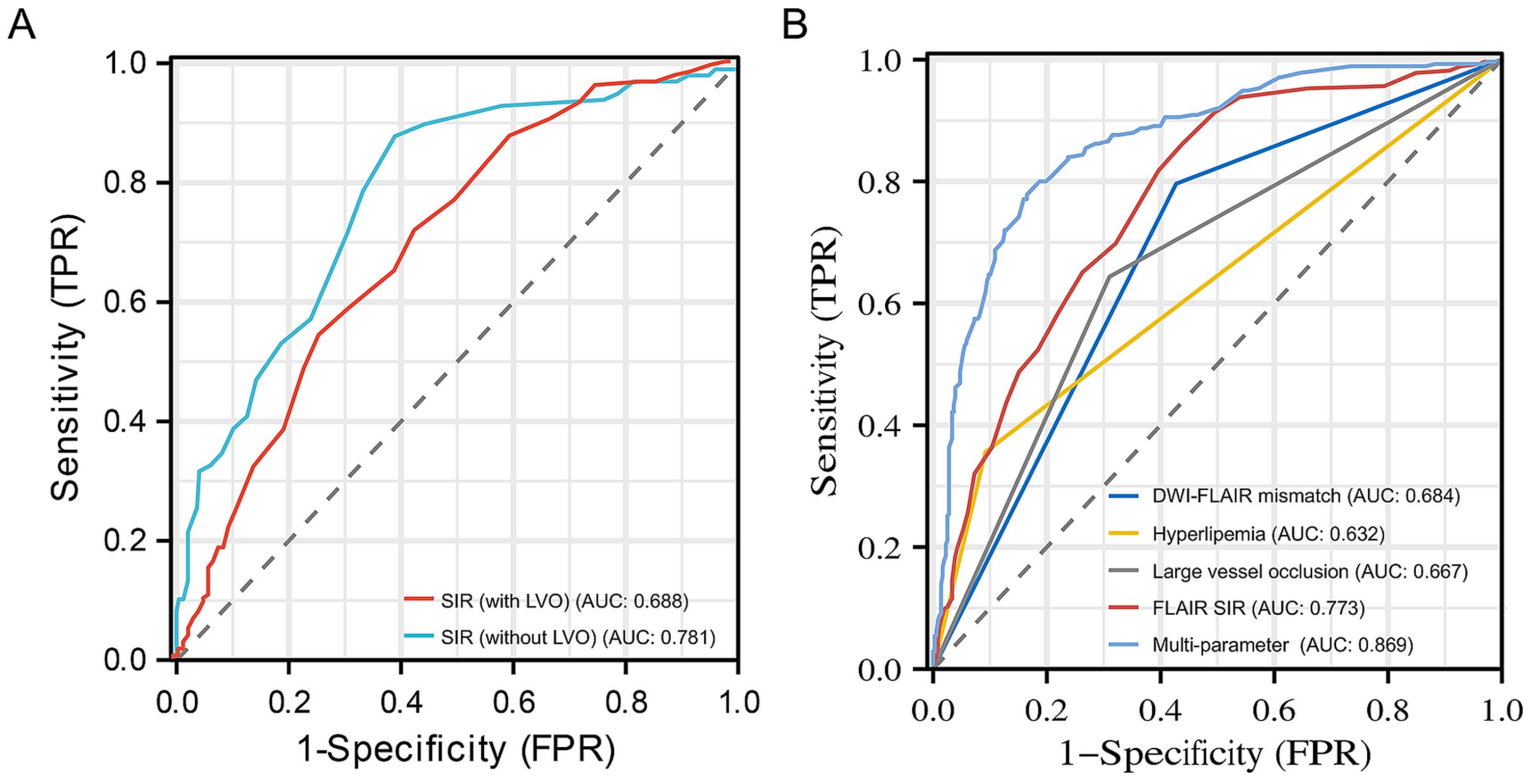
Receiver operating characteristic curves for SIR with or without LVO (A) and different parameters (B) for identifying stroke within 4.5 h. The 0.781 AUC of SIR without LVO was higher than 0.688 AUC of SIR with LVO in identifying stroke within 4.5 h. The 0.869 AUC of the multiparameter model (hyperlipemia, LVO and SIR) showed significantly improved performance in identifying stroke within 4.5 h over that of the DWI-FLAIR mismatch (0.684), hyperlipemia (0.632), large vessel occlusion (0.667) and SIR (0.773) models. DWI, diffusion-weighted imaging; FLAIR, fluid-attenuated inversion recovery; SIR, signal intensity ratio; AUC, area under curve; LVO, large vessel occlusion.

In identifying stroke within 4.5 h, the DWI-FLAIR mismatch model achieved an AUC of 0.684 (95% CI: 0.649–0.720), the SIR model achieved an AUC of 0.773 (95% CI: 0.737–0.809), the hyperlipemia model had an AUC of 0.632 (95% CI: 0.600–0.664), and the LVO model achieved an AUC of 0.667 (95% CI: 0.630–0.704). The SIR model showed significantly better performance than the DWI-FLAIR mismatch model (*z* = −3.587; *p* < 0.001). The multiparameter model (including hyperlipemia, LVO and SIR) yielded an AUC of 0.869 (95% CI: 0.841–0.897), a significant performance improvement over the DWI-FLAIR mismatch (*z* = −8.565; *p* < 0.001), SIR (*z* = −6.259, *p* < 0.001), hyperlipemia (*z* = −13.565, *p* < 0.001) and LVO (*z* = −11.956, *p* < 0.001) models. The performance of the models is shown in Table 4. ROC curves for different parameters for identifying the onset time is shown in Figure 4. When the threshold of multiparameter model was ≤0.470, the specificity and sensitivity for predicting stroke within 4.5 h was 0.77 and 0.835, respectively.

**Table 4:** The performance of different variables in identifying the time from stroke onset.

	AUC	Cutoff	Accuracy	Sensitivity	Specificity	PPV	NPV
DWI-FLAIR mismatch	0.684 (0.649–0.720)	0.5	0.670	0.796	0.573	0.589	0.785
Hyperlipemia	0.632 (0.600–0.664)	0.5	0.668	0.356	0.908	0.748	0.647
Large artery occlusion	0.667 (0.630–0.704)	0.5	0.669	0.643	0.690	0.615	0.716
SIR	0.773 (0.737–0.809)	1.115	0.692	0.862	0.561	0.602	0.841
SIR (without LVO)	0.781 (0.727–0.834)	1.105	0.687	0.876	0.611	0.473	0.926
SIR (with LVO)	0.688 (0.624–0.752)	1.145	0.660	0.718	0.568	0.726	0.558
Multi-parameters	0.869 (0.841–0.897)	0.470	0.810	0.778	0.835	0.784	0.831

Multi-parameters including hyperlipemia, large artery occlusion and SIR. AUC, area under the curve; PPV, positive predictive value; NPV, negative predictive value; DWI, diffusion-weighted imaging; FLAIR, fluid attenuated inversion recovery; SIR, signal intensity ratio; LVO, large vessel occlusion.

## Discussion

In this retrospective multicenter cohort study, we found that patients with a time from symptom onset to MRI >4.5 h had a higher rate of hyperlipemia, almost double the rate of LVO and a higher SIR than those within 4.5 h. The SIR was positively correlated with the onset time in patients both with and without LVO. LVO partially mediated the relationship between SIR and stroke within 4.5 h. In addition, we found that the SIR model performed significantly better than the DWI-FLAIR mismatch model in identifying stroke within 4.5 h, and a model that included hyperlipemia, LVO and the SIR was the best.

Intracerebral postischemia cytotoxic edema reduces water diffusivity and is detected on brain MRI as a hyperintensity on DWI with corresponding low ADC values (20). Over time, the manifestations of vascular edema become evident on FLAIR images, gradually exhibiting a high signal intensity (21). Consequently, by discerning the temporal disparity in imaging, the exact onset time can be feasibly ascertained (22). Our analysis demonstrated that DWI-FLAIR mismatch can qualitatively identify stroke within 4.5 h with high sensitivity. Thomalla et al. (23) reported that stroke lesions detected with DWI-FLAIR mismatch are likely to be within a time window where intravenous tissue plasminogen activator can be administered safely. However, we found that DWI-FLAIR mismatch has low specificity and, as such, cannot be used alone to guide acute treatment decision-making. Although this method is direct, it is highly subjective, which makes consistency of the results difficult (24). This qualitative observation underscores the significance of employing quantitative assessments with the SIR, particularly in cases where alterations are nuanced. The FLAIR SIR calculation can be efficiently performed on an MRI console during emergent stroke scenarios, and research has demonstrated that this quantitative FLAIR analysis is more precise in identifying acute strokes than relying solely on qualitative DWI-FLAIR mismatch. In our study, we observed that the SIR value could identify strokes within 4.5 h, with a sensitivity of 0.862 and a specificity of 0.561. These findings are in accordance with those of Legge et al. (15), who calculated the FLAIR SIR by manually delineating ROIs to identify stroke onset within 4.5 h, with a sensitivity of 0.83 and a specificity of 0.67. However, our method utilized quantitative methods to determine the ROI and is therefore more objective and reproducible.

This quantitative SIR evaluation can be particularly important when the changes on FLAIR imaging are subtle. It also indicates that there are other factors in addition to time that can affect FLAIR SI (25). Our analysis demonstrated that patients with onset time >4.5 h had a greater incidence of hyperlipemia, almost double the prevalence of LVO and a greater SIR than those with onset time ≤4.5 h. In a non-randomized study design, the two groups may exhibit disparities in certain baseline characteristics. The observed higher incidence of hyperlipidemia and LVO in the onset time >4.5 h group could potentially be attributed to these baseline difference. In addition, we found that the SIR was positively correlated with the symptom onset to MRI time in patients both with and without LVO, and mediation analysis revealed that LVO partially mediated the relationship between SIR and the onset time. These findings are also the innovations of this study. The elevation of blood lipids in patients facilitates their deposition within blood vessels, resulting in inflammatory responses within the intima of the vascular wall (26). This process subsequently leads to the proliferation of fibrous tissue, gradually diminishing the elasticity of the arteries and promoting its thickening. Narrowing of the blood vessels and the development of atherosclerosis indirectly contribute to vascular obstruction (27, 28). Hyperlipidemia has the potential to modify the water molecule environment in adjacent tissues, resulting in an increase in the length of the transverse relaxation time of water molecules. Consequently, this elongation enhances the T2 effect, ultimately leading to an augmentation of FLAIR signals. This may partly explain the effect of hyperlipemia and LVO on the SIR and onset time. In addition, we found that LVO partially mediated the relationship between SIR and the onset time. The occlusion of large blood vessels can result in the cessation of blood circulation, leading to inadequate delivery of oxygen and nutrients to cerebral tissue (29, 30). Consequently, a progressive expansion of stroke-related damage occurs as cellular death intensifies (31). Civrny et al. (12) made the following hypothesis based on their research results that acute stroke patients with LVO exhibited a poorer collateral circulation supply, resulting in an accelerated progression of signal changes in the ischemic core area compared to patients without LVO. The size of the ischemic lesion is a recognized determinant influencing the relationship between the FLAIR SIR and the onset time (32). Larger ischemic lesions frequently exhibit heightened FLAIR SI due to cellular damage and death, resulting in fluid movement within and outside the cells. This fluid displacement elevates the water molecule content in the adjacent brain tissue, thereby manifesting as an augmented FLAIR SI.

On the basis of the above characterizations, we further constructed a multiparameter model for identifying stroke within 4.5 h. We found that the multiparameter model yielded an AUC of 0.869, reflecting a significant performance improvement over the DWI-FLAIR mismatch, SIR, hyperlipemia and LVO models. Taner Bulut et al. (10) demonstrated that FLAIR SIR threshold of ≤1.18 for predicted symptom onset 4.5 h increased specificity (0.77 vs. 0.74) and sensitivity (0.77 vs. 0.69) as compared with visual analysis. FLAIR SIR threshold of ≤1.19 for predicted symptom onset 6 h increased sensitivity (0.76 vs. 0.67) and equal specificity (0.75 vs. 0.75) as compared with visual analysis. Our results indicated that the AUC for SIR in predicting onset time is 0.773, which consistent with prior studies. However, the specificity remains relatively low at 0.561. The low specificity can be attributed to the diminished capability of SIR to accurately identify patients within 4.5 h of symptom onset. This limitation may stem from the observation that some patients within this time frame do not exhibit pronounced hyperintensity on FLAIR imaging. Furthermore, the FLAIR signal intensity is subject to inter-patient variability. Additionally, the onset time of stroke is influenced by a multitude of factors, rendering the reliance on SIR alone for predicting onset time both imprecise and nonspecific. It is worth noting that our multiparameter model can more accurately identify an onset time within 4.5 h, with a sensitivity of 0.778 and a specificity of 0.835 when the threshold was ≤0.470. This discovery holds great importance, particularly when considering the favorable and promising outcomes observed in recent trials involving intra-arterial tissue plasminogen activator.

Our study has several limitations. First, we calculated the SI based on ROIs rather than voxels, which can lead to a more accurate value. Second, we ensured a sample balance between patients with an onset time below 4.5 h and above 4.5 h, and thus bias could not be avoided. However, the large sample size in this study reduces this potential bias. Finally, strokes were not subjected to meticulous analysis regarding their subtype etiologies or the specific hemisphere of the brain in which they occurred. Conducting a more thorough examination of the stroke subtype and the affected hemisphere could enhance our comprehension of the SIR as a proxy for determining the onset time.

## Conclusion

In conclusion, quantitative assessment of the FLAIR SIR can be used as a proxy for lesion age in patients with WUS or an unknown onset time. The SIR and stroke within 4.5 h were partially mediated by LVO. The multiparameter model combines hyperlipemia, LVO and SIR and can more accurately identify the time from stroke onset.

## Data Availability

The data supporting the findings of this study may be requested from the corresponding author.
